# Improving Our Understanding of *Salmonella enterica* Serovar Paratyphi B through the Engineering and Testing of a Live Attenuated Vaccine Strain

**DOI:** 10.1128/mSphere.00474-18

**Published:** 2018-11-28

**Authors:** Ellen E. Higginson, Girish Ramachandran, Tracy H. Hazen, Dane A. Kania, David A. Rasko, Marcela F. Pasetti, Myron M. Levine, Sharon M. Tennant

**Affiliations:** a Center for Vaccine Development and Global Health, University of Maryland School of Medicine, Baltimore, Maryland, USA; b Department of Medicine, University of Maryland School of Medicine, Baltimore, Maryland, USA; c Institute for Genome Sciences, Department of Microbiology and Immunology, University of Maryland School of Medicine, Baltimore, Maryland, USA; d Department of Pediatrics, University of Maryland School of Medicine, Baltimore, Maryland, USA; U.S. Food and Drug Administration

**Keywords:** Paratyphi B, *Salmonella*, genomics, live attenuated, oral vaccines

## Abstract

Enteric fever is caused by three Salmonella enterica serovars: Typhi, Paratyphi A, and Paratyphi B *sensu stricto*. Although vaccines against two of these serovars are licensed (Typhi) or in clinical development (Paratyphi A), as yet there are no candidates for *S.* Paratyphi B. To gain genomic insight into these serovars, we sequenced 38 enteric fever-associated strains from Chile and compared these with reference genomes. Each of the serovars was separated genomically based on the core genome. Genomic comparisons identified loci that were aberrant between serovars Paratyphi B *sensu stricto* and Paratyphi B Java, which is typically associated with gastroenteritis; however, the majority of these were annotated as hypothetical or phage related and thus were not ideal vaccine candidates. With the genomic information in hand, we engineered a live attenuated *S.* Paratyphi B *sensu stricto* vaccine strain, CVD 2005, which was capable of protecting mice from both homologous challenge and heterologous challenge with *S.* Paratyphi B Java. These findings extend our understanding of *S.* Paratyphi B and provide a viable vaccine option for inclusion in a trivalent live attenuated enteric fever vaccine formulation.

**IMPORTANCE** We developed a live attenuated Salmonella enterica serovar Paratyphi B vaccine that conferred protection in mice against challenge with *S*. Paratyphi B *sensu stricto* and *S*. Paratyphi B Java, which are the causes of enteric fever and gastroenteritis, respectively. Currently, the incidence of invasive *S.* Paratyphi B *sensu stricto* infections is low; however, the development of new conjugate vaccines against other enteric fever serovars could lead to the emergence of *S.* Paratyphi B to fill the niche left by these other pathogens. As such, an effective *S.* Paratyphi B vaccine would be a useful tool in the armamentarium against Salmonella infections. Comparative genomics confirmed the serovar-specific groupings of these isolates and revealed that there are a limited number of genetic differences between the *sensu stricto* and Java strains, which are mostly hypothetical and phage-encoded proteins. The observed level of genomic similarity likely explains why we observe some cross-protection.

## Abstract (precis)

We developed a live attenuated Salmonella enterica serovar Paratyphi B vaccine that conferred protection in mice against challenge with *S*. Paratyphi B *sensu stricto* and *S*. Paratyphi B Java, which are the causes of enteric fever and gastroenteritis, respectively. Currently, the incidence of invasive *S.* Paratyphi B *sensu stricto* infections is low; however, the development of new conjugate vaccines against other enteric fever serovars could lead to the emergence of *S.* Paratyphi B to fill the niche left by these other pathogens. As such, an effective *S.* Paratyphi B vaccine would be a useful tool in the armamentarium against Salmonella infections. Comparative genomics confirmed the serovar-specific groupings of these isolates and revealed that there are a limited number of genetic differences between the *sensu stricto* and Java strains, which are mostly hypothetical and phage-encoded proteins. The observed level of genomic similarity likely explains why we observe some cross-protection.

## INTRODUCTION

Enteric fever is a major cause of morbidity and mortality worldwide, with recent estimates suggesting that there are ∼13.5 million cases (1), and 217,000 deaths (2) each year. The descriptor “enteric fever” refers to two separate, but clinically indistinguishable infectious diseases: typhoid fever and paratyphoid fever. Although typhoid fever has historically shown the greatest prevalence, recent epidemiological surveillance has revealed an increase in paratyphoid A fever across parts of southeast and central Asia (3–5). As such, there is great interest in a vaccine that can protect against both typhoid fever and paratyphoid fever.

Three licensed vaccines currently exist for typhoid fever: the unconjugated Vi polysaccharide vaccine, Vi conjugate vaccine, and Ty21a live attenuated vaccine (6). While the Vi-based vaccines cannot confer cross-protection against paratyphoid strains, in large-scale, randomized controlled field trials of Ty21a live oral vaccine in Santiago, Chile, a moderate level of cross-protection was conferred by Ty21a vaccination against *S.* Paratyphi B disease (7–9). In a second field trial of Ty21a in Indonesia, where both Salmonella enterica serovars Typhi and Paratyphi A were circulating, Ty21a protected against typhoid but not against paratyphoid A fever (10). Furthermore, in clinical studies, individuals immunized with Ty21a produced moderate antibody responses that cross-reacted with both *S.* Paratyphi B and *S.* Paratyphi A (11, 12). Multifunctional CD8^+^ T cell responses were also elicited in Ty21a-immunized volunteers, which recognized *S.* Typhi, and to a lesser extent *S.* Paratyphi B, but not *S.* Paratyphi A (13). These data suggest that additional or different vaccines are required for adequate protection against paratyphoid fever. There are multiple vaccines in clinical development for *S*. Paratyphi A, including a live attenuated vaccine (ATCC 9150 Δ*guaBA* Δ*clpX* [CVD 1902]) and several conjugate vaccines consisting of the *S.* Paratyphi A O polysaccharide (OPS) (O:2,12) conjugated to different immunogenic proteins, such as tetanus toxoid, diphtheria toxoid, and CRM_197_, a nontoxic mutant of diphtheria toxin (15).

Unlike for *S*. Typhi and *S.* Paratyphi A, no vaccine candidates exist for *S.* Paratyphi B. There are two variants of *S.* Paratyphi B, Java and *sensu stricto*, which are differentiated based on their ability to ferment d-tartrate (the *sensu stricto* variant is d-tartrate fermentation negative, and Java is d-tartrate fermentation positive) (16, 17). Strains of the Java lineage have traditionally been associated with gastroenteritis, while *sensu stricto* strains cause enteric fever (16). In a recent study, the core genomes of 191 *S.* Paratyphi B isolates were compared to better understand the genetic composition of this serovar (18). The strains clustered into 10 distinct phylogroups (PGs), with PG1 containing all of the classical *sensu stricto* strains. Java strains were split across PG2 to -10, with PG2 to -5 harboring some strains that were isolated from patients with invasive disease. Except for clinical presentation, there are few data describing the similarities and differences between the three enteric fever serovars.

*S.* Paratyphi B *sensu stricto* infections are presently uncommon; however, in previous decades they constituted a significant proportion of disease. In the 1980s when typhoid was hyperendemic in Santiago, Chile, *S.* Paratyphi B *sensu stricto* had an incidence rate of 71.1 cases per 10^5^ school age children over 3 years of follow-up, about one-fifth the incidence of *S.* Typhi among school age children at the time (9). Although the incidence of invasive *S.* Paratyphi B *sensu stricto* infections is currently low, the availability of *S.* Typhi vaccines, as well as the development of novel *S.* Paratyphi A vaccines, could create a niche for *S*. Paratyphi B in the absence of these other pathogens. The availability of an efficacious *S.* Paratyphi B vaccine would therefore be a useful weapon to add to the growing arsenal against Salmonella infections. The aim of this study was to develop a live attenuated vaccine that can protect against *S.* Paratyphi B disease and in doing so learn more about this serovar through whole-genome sequencing and evaluation of virulence in mice.

## RESULTS

### Comparative genomic analysis of typhoidal *Salmonella* strains from Chile.

**FIG 1:**
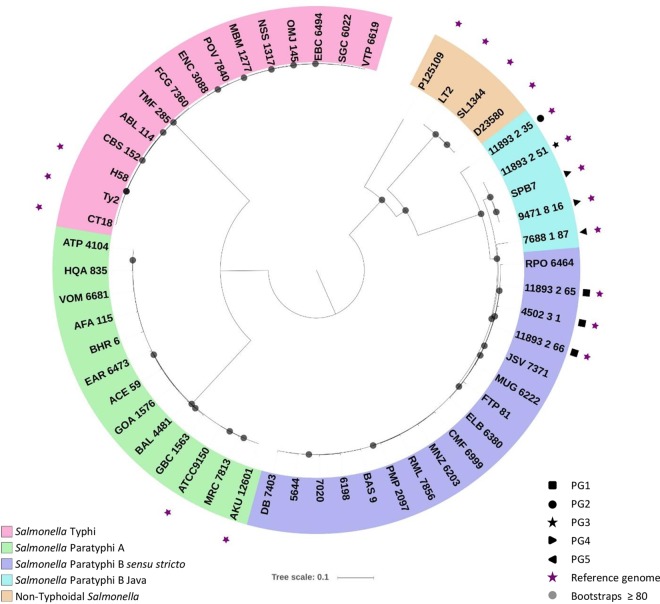
Phylogenetic analysis of typhoidal Salmonella strains isolated in Chile. Genomes from bacterial strains isolated in Chile were compared with Salmonella reference strains. Phylogenies were produced using the In Silico Genotyper pipeline (41), using SL1344 as a reference and the tree file generated in RaxML (42). Tree images were created using iTOL (43).

Genome sequencing was completed on 14 *S.* Paratyphi B *sensu stricto* strains and 11 *S.* Paratyphi A and 12 *S.* Typhi strains isolated in Chile between 1983 and 1986 (see Table S1 in the supplemental material). A single nucleotide polymorphism (SNP)-based conserved core genome analysis confirmed that Chilean *S.* Paratyphi B isolates clustered with *S.* Paratyphi B reference strains (isolated in the 2000s) designated phylogroup 1 (PG1) by Connor et al. (Fig. 1). Additionally, each of the other serogroups mapped to the included reference isolates for that serogroup. These results indicate that the Salmonella isolates from Chile are not significantly different from other Salmonella isolates.

10.1128/mSphere.00474-18.2 TABLE S1 Salmonella strains used for whole-genome sequencing and analysis. Download Table S1, DOCX file, 0.01 MB. Copyright © 2018 Higginson et al. 2018 Higginson et al. This content is distributed under the terms of the Creative Commons Attribution 4.0 International license.

The whole-genome content of sequenced isolates was compared at the gene level by large-scale BLAST score ratio (LS-BSR) analysis, which compares the gene contents of all included genomes. Sixty-one gene features (centroids) were present in *S*. Paratyphi B *sensu stricto* strains but absent from *S*. Paratyphi B Java (see Table S2 in the supplemental material). These gene features were primarily located in four genomic loci, containing mostly hypothetical proteins and predicted phage proteins. In the converse analysis, 43 gene features were present in three genomic loci in *S.* Paratyphi B Java strains but absent in the *sensu stricto* strains (see Table S3 in the supplemental material). Several of these gene features encoded putative multidrug transporters. While core and accessory genomic differences could be identified between the serovars, they were not appropriate targets for vaccine development.

10.1128/mSphere.00474-18.3 TABLE S2 Large-scale BLAST score ratios (LS-BSR) for genes conserved in Salmonella Paratyphi B *sensu stricto* (>0.8) but absent in Salmonella Paratyphi B Java (<0.4). Download Table S2, XLSX file, 0.02 MB. Copyright © 2018 Higginson et al. 2018 Higginson et al. This content is distributed under the terms of the Creative Commons Attribution 4.0 International license.

10.1128/mSphere.00474-18.4 TABLE S3 Large-scale BLAST score ratios (LS-BSR) for genes conserved in Salmonella Paratyphi B Java (>0.8) but absent in Salmonella Paratyphi B *sensu stricto* (<0.4). Download Table S3, XLSX file, 0.02 MB. Copyright © 2018 Higginson et al. 2018 Higginson et al. This content is distributed under the terms of the Creative Commons Attribution 4.0 International license.

### Mouse infection model for *Salmonella* paratyphi B.

Mouse infection models were developed for both *S.* Paratyphi B *sensu stricto* and Java. BALB/c mice (*n* = 3 per strain and infection route) were infected with 10^8^ CFU of *S.* Paratyphi B *sensu stricto* (strain CMF 6999, ELB 3830, or JSV 7371) or Java (strain CDC00-0301, CDC01-0516, or CDC03-0451), via either the peroral (p.o.) or intraperitoneal (i.p.) route. All mice infected via the i.p. route, succumbed to the infection within 24 h (Table 1). In contrast, the majority of mice survived p.o. challenge. Due to the greater lethality, the i.p. route was chosen for further analysis. As there was no significant difference in virulence between the strains, CMF 6999 (*sensu stricto*) and CDC00-0301 (Java) were selected for further analysis. The 50% lethal dose (LD_50_) of *S.* Paratyphi B *sensu stricto* CMF 6999 for the i.p. route was subsequently determined to be 1.4 × 10^6^ CFU, while the LD_50_ for *S.* Paratyphi B Java CDC00-0301 was 8 × 10^5^ CFU (Table 2).

**TABLE 1:** Lethality of *Salmonella* Paratyphi B strains in mice

Serovar	Strain	No. died/tested by route:	
		p.o.	i.p.
*S.* Paratyphi B *sensu stricto*	JSV 7371	1/3	3/3
	ELB 6380	1/3	3/3
	CMF 6999	0/3	3/3
*S.* Paratyphi B Java	CDC00-0301	0/3	3/3
	CDC01-0516	0/3	3/3
	CDC03-0451	0/3	3/3

**TABLE 2:** LD_50_ values for bacterial strains via the i.p. route

Strain	Variant	Genotype	LD_50_ (CFU)
CMF 6999	*Sensu stricto*	Wild type	1.4 × 10^6^
CDC00-0301	Java	Wild type	8.0 × 10^5^
CVD 2003	*Sensu stricto*	CMF 6999 Δ*guaBA*	>5 × 10^8^
CVD 2004	*Sensu stricto*	CMF 6999 Δ*clpX*	4.25 × 10^7^
CVD 2005	*Sensu stricto*	CMF 6999 Δ*guaBA* Δ*clpX*	>5 × 10^8^

### Construction of vaccine strains and phenotypic characterization.

For the construction of a live attenuated Salmonella Paratyphi B vaccine strain, we chose to target two known attenuating mutations, *guaBA* and *clpX*. Mutations in these genes have previously been used in vaccines for other Salmonella serovars, including Typhimurium and Enteritidis (19) and Paratyphi A (14). Importantly, the *S*. Paratyphi A live attenuated vaccine CVD 1902 (Δ*guaBA* Δ*clpX*) was found to be well tolerated in a phase 1 clinical trial. The *guaBA* locus encodes guanine biosynthesis, while *clpPX* encodes a protease that degrades the master flagellar regulator FlhD/FlhC (20, 21), resulting in increased motility. Mutations were introduced into Salmonella Paratyphi B *sensu stricto* strain CMF 6999, creating strains CVD 2003 (Δ*guaBA*), CVD 2004 (Δ*clpX*), and CVD 2005 (Δ*guaBA* Δ*clpX*).

To confirm the predicted mutant phenotypes, strains were subjected to targeted phenotypic tests. Mutation of the *guaBA* locus leads to guanine auxotrophy. To confirm the deletion and subsequent complementation of *guaBA* mutants, strains were grown on chemically defined medium, with or without the addition of exogenous guanine. Results are shown in Table S4 in the supplemental material. As expected, both CVD 2003 (Δ*guaBA*) and CVD 2005 (Δ*guaBA* Δ*clpX*) were unable to grow on chemically defined medium lacking guanine. Growth was restored by addition of guanine to the medium or by complementation of *guaBA* on a plasmid.

10.1128/mSphere.00474-18.5 TABLE S4 Guanine auxotrophy of *guaBA* mutant strains. Download Table S4, DOCX file, 0.01 MB. Copyright © 2018 Higginson et al. 2018 Higginson et al. This content is distributed under the terms of the Creative Commons Attribution 4.0 International license.

For *clpX* mutants, strains were assessed for changes in motility. The ClpX protein negatively regulates flagellar expression in *S.* Typhimurium (20); hence, Δ*clpX* mutants were predicted to have increased numbers of flagella and motility. Both the CVD 2004 (Δ*clpX*; 20.0 ± 1.5 mm [mean ± standard deviation]) and CVD 2005 (Δ*guaBA* Δ*clpX*; 22.6 ± 0.7 mm) mutants showed significantly increased zones of motility over wild-type strain CMF 6999 (13.8 ± 1.9 mm) on motility agar (*P* = 0.032 and *P* = 0.0021, respectively) (see Fig. S1 in the supplemental material). The increase in motility by flagellar overexpression could be restored to wild-type levels by the addition of the *clpX* gene in *trans* (Fig. S1).

10.1128/mSphere.00474-18.1 FIG S1 Motility of *S*. Paratyphi B Δ*clpX* mutant strains. Bacterial strains from overnight cultures were inoculated onto motility agar plates by the use of a straight wire. Plates were incubated for 18 h at 37°C, and the zones of motility were measured. Bars: 1, CMF 6999(pLowBlu); 2, CVD 2004(pLowBlu); 3, CVD 2004(pATGclpX); 4, CVD 2005(pLowBlu); 5, CVD 2005(pATGguaBAATGclpX). Data are shown as mean ± standard deviation (SD). *, *P* < 0.05, and **, *P* < 0.01, Student’s *t* test. Download FIG S1, PDF file, 0.3 MB. Copyright © 2018 Higginson et al. 2018 Higginson et al. This content is distributed under the terms of the Creative Commons Attribution 4.0 International license.

### Attenuation of strains in the mouse model.

The 50% lethal dose for each of the CMF 6999 mutant strains was determined by challenging BALB/c mice i.p. with *S.* Paratyphi B strains at doses ranging from 10^3^ to 10^9^ CFU (Table 2). All three mutant strains were attenuated, with the LD_50_ for strain CVD 2004 1 log_10_ greater than that of the wild-type strain and those of CVD 2003 and CVD 2005 greater than 2 log_10_ higher than that of the wild type.

### Immunization of mice with strain CVD 2005.

**FIG 2:**
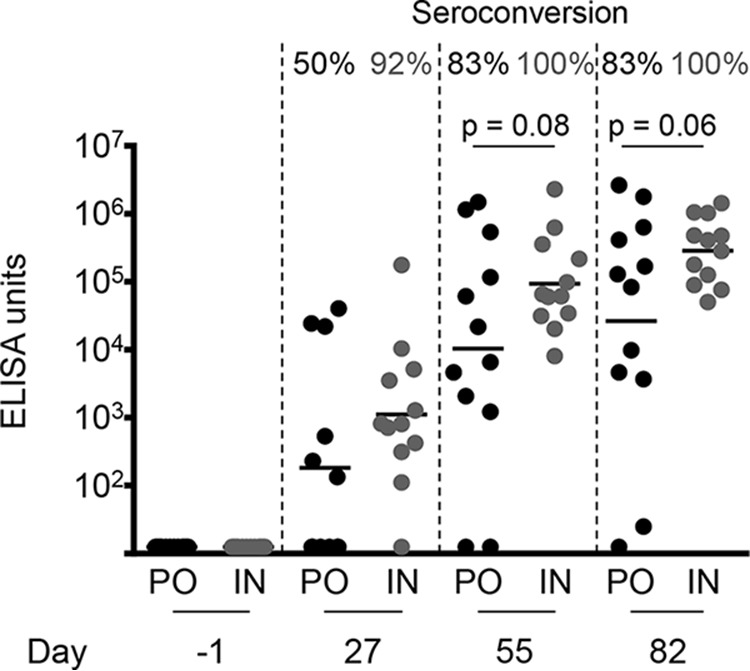
Serum IgG responses to LPS from mice immunized with CVD 2005 (*S.* Paratyphi B *sensu stricto* CMF 6999 Δ*guaBA* Δ*clpX*). Mice were administered three doses of vaccine CVD 2005 on days 0, 28, and 56. Sera were collected from mice on days −1, 27, 55, and 82. Antibody responses to LPS were assessed by ELISA, using purified O serogroup B polysaccharide. Each point represents an individual mouse, with the average shown as the geometric mean titer. The statistical significance (Student's *t* test) and percentage of seroconversion are shown above. PO, peroral; IN, intranasal.

Groups of 12 mice were immunized either p.o. or intranasally (i.n.) with three doses of 10^9^ CFU of CVD 2005 28 days apart. The vaccine was well tolerated, with no noticeable side effects following immunization. The antibody responses to the vaccine were assessed by measuring lipopolysaccharide (LPS)-specific serum IgG titers by enzyme-linked immunosorbent assay (ELISA). An increase in IgG levels was observed after each immunization by either route (Fig. 2). There was no significant difference between the two routes in either geometric mean titer or seroconversion.

**FIG 3:**
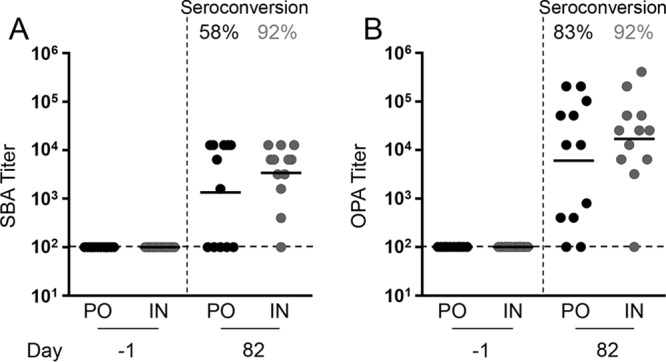
Serum bactericidal antibody (SBA) and opsonophagocytosis antibody (OPA) assays on sera collected from CVD 2005 (*S.* Paratyphi B *sensu stricto* CMF 6999 Δ*guaBA* Δ*clpX*)*-*immunized mice. Immune serum was incubated with early-log-phase-grown bacteria and baby rabbit complement for 1 h. For OPA assays, HL60 cells were added after a 15-min preincubation. Bacterial survival after 1 h was determined by plate counts. (A) SBA against *S*. Paratyphi B CMF 6999. (B) OPA against *S*. Paratyphi B CMF 6999. The titer was defined as the highest dilution of sera that could deliver greater than 50% complement killing. Each point represents one mouse. Dashed lines represent the limit of detection. The geometric mean and percentage of seroconversion are shown for each immunized group. PO, peroral; IN, intranasal.

Functional activity of the antibodies was assessed using serum bactericidal antibody (SBA) and opsonophagocytic antibody (OPA) assays. The SBA assay measures complement-mediated killing, while the OPA assay measures the ability of antibodies to facilitate opsonophagocytosis. Both routes induced high functional antibody responses. There was no significant difference in overall titer or the rate of seroconversion between the two dosage routes for either the SBA (Fig. 3A) or the OPA (Fig. 3B) assays.

### Challenge of vaccinated mice with *S.* Paratyphi B *sensu stricto* and Java and *S.* Typhimurium.

**FIG 4:**
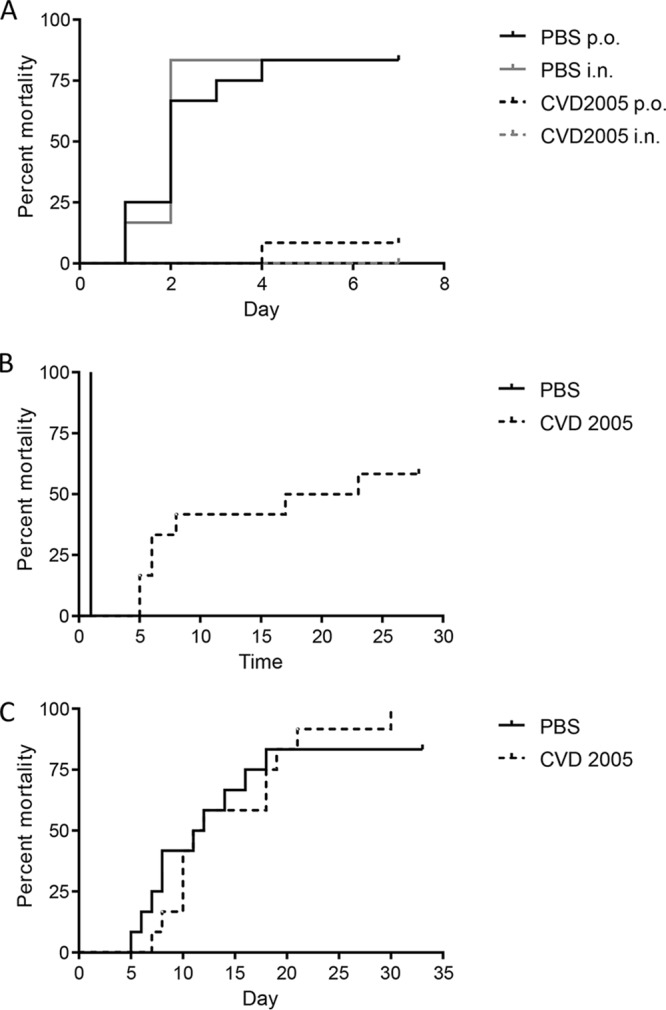
Cumulative mortality for CVD 2005-immunized mice after challenge with homologous and heterologous serovars. Mice immunized with either PBS or CVD 2005 (*S.* Paratyphi B *sensu stricto* CMF 6999 Δ*guaBA* Δ*clpX)* were challenged with 2 × 10^7^ CFU of *S.* Paratyphi B *sensu stricto* strain CMF 6999 (A), *S.* Paratyphi B Java CDC00-0301 (B) via the i.p. route, or *S.* Typhimurium I77 (C) via the p.o. route. Mice were monitored for up to 33 days.

To determine vaccine efficacy, immunized mice were challenged i.p. on day 82 with *S.* Paratyphi B *sensu stricto* strain CMF 6999. For mice receiving phosphate-buffered saline (PBS), there was an 83% (10/12) mortality rate (Fig. 4A). Animals succumbed to infection within 1 to 4 days postchallenge, with a mean time to death of 2 days. In contrast, mice that received CVD 2005 via either the i.n. or p.o. route were significantly protected, with an 8% (1/12) mortality rate observed among p.o.-immunized mice and zero mortality for i.n.-immunized mice. This corresponded to vaccine efficacies of 90% for p.o. immunization and 100% for i.n. immunization against challenge with the homologous serovar (Table 3).

**TABLE 3:** Vaccine efficacy of strain CVD 2005 against lethal challenge

Immunization route	Immunization	Challenge^*a*^	Mortality rate	Time to death		Vaccine efficacy	
				Mean (days)	*P* value (Student’s *t* test)	% efficacy	*P* value (Fisher’s exact test)
p.o.	PBS	*Sensu stricto*	10/12	2	NS^*b*^	90	<0.001
	CVD 2005	*Sensu stricto*	1/12	4			

i.n.	PBS	*Sensu stricto*	10/12	2	NA^*c*^	100	<0.001
	CVD 2005	*Sensu stricto*	0/12	NA			
	PBS	Java	12/12	1	0.016	42	0.037
	CVD 2005	Java	7/12	10			
	PBS	Typhimurium	10/12	11	NS	0	NS
	CVD 2005	Typhimurium	12/12	15			

a Represented are *S.* Paratyphi B *sensu stricto* strain CMF 6999, *S.* Paratyphi B Java strain CDC00-0301, and *S.* Typhimurium strain I77.

b NS, not significant (*P* > 0.05).

c NA, not applicable.

In a heterologous challenge with *S*. Paratyphi B Java, 100% of mice receiving PBS rapidly succumbed to infection (Fig. 4B). Immunized mice exhibited a 58% mortality rate. Although vaccine efficacy was modest (42%), the mean time to death was significantly longer in immunized mice (Table 3 and Fig. 4B). We also challenged CVD 2005-immunized mice perorally with *S.* Typhimurium, which shares the same O antigens as *S*. Paratyphi B (1,4,[5],12). We observed no vaccine efficacy against low (LD_50_ of ∼100) *S.* Typhimurium challenge (Fig. 4C).

## DISCUSSION

We performed whole-genome sequencing to achieve two goals. First, analysis of conserved genome SNPs confirmed that the *S.* Paratyphi B *sensu stricto* strains from Chile cluster with other invasive disease strains. In particular, the *S*. Paratyphi B *sensu stricto* strains (including the parental strain of our live attenuated vaccine) isolated in Chile in the 1980s cluster with *S*. Paratyphi B *sensu stricto* strains isolated in the 2000s. This demonstrates that our live vaccine strain is genetically similar to modern *S*. Paratyphi B *sensu stricto* isolates and could therefore confer full protection against currently circulating strains. Second, the comparative genomics analysis revealed that there are a limited number of genetic loci separating the *sensu stricto* and Java strains, and their products are mostly hypothetical and phage-encoded proteins. The observed level of genomic similarity probably explains why these isolates behave similarly in mice. The subtypes do, however, present differently clinically in human infections, which will require further analysis.

Through the engineering of a live attenuated vaccine for *S*. Paratyphi B, we have been able to make numerous observations relating to the pathogenesis of this serovar and its relationship with other enteric fever-associated serovars. While many nontyphoidal Salmonella strains readily infect mice orally, *S.* Typhi and *S*. Paratyphi A do not typically cause disease in mice unless given intraperitoneally with hog gastric mucin (22–25). The molecular or cellular bases as to why hog gastric mucin is necessary for a productive infection in mice are not known. *S.* Paratyphi B *sensu stricto* strains seem to have an intermediate phenotype between these two extremes, being lethal for mice intraperitoneally in the absence of hog gastric mucin, but not lethal orally. This conforms to the theory that *S.* Paratyphi B is less human host restricted than other enteric fever serovars. In the comparative genomic analysis, we noted many differences in genes related to bacterial metabolism (see Table S5 in the supplemental material). Metabolic gene loss is a characteristic of *S.* Typhi and *S*. Paratyphi A and is thought to be one of the means by which they became exquisitely host adapted (26). *S.* Paratyphi B in contrast has maintained many of these metabolic loci, which may in part explain why they are more virulent in mice.

10.1128/mSphere.00474-18.6 TABLE S5 Large-scale BLAST score ratios (LS-BSR) for genes conserved in Salmonella Paratyphi B (*sensu stricto* and Java [>0.8]) but absent in Salmonella Typhi and Paratyphi A (<0.4). Download Table S5, XLSX file, 0.05 MB. Copyright © 2018 Higginson et al. 2018 Higginson et al. This content is distributed under the terms of the Creative Commons Attribution 4.0 International license.

As with other Salmonella serovars (14, 19), both the Δ*guaBA* and Δ*clpX* mutations were attenuating in *S.* Paratyphi B. Vaccine strain CVD 2005 was well tolerated and protective in mice against both homologous and heterologous challenge. Upon mucosal immunization, strong humoral responses were observed in vaccinated mice regardless of the route of vaccination. This is consistent with other Salmonella live attenuated vaccines administered to mice, including the live attenuated vaccine strains *S.* Typhi Ty21a, CVD 915, and CVD 908 *htrA*, and *S.* Paratyphi A vaccine candidate CVD 1901 (25, 27). While not significant, there was a trend toward increased antibody titer and functional capacity in i.n.-immunized mice over those receiving p.o. administration. Intranasal immunization allows for direct access of vaccine organisms to the immune cells of the nasal-associated lymphoid tissue and is known to be more immunogenic than oral vaccination in mice (28).

*S.* Paratyphi B Java is a common cause of gastroenteritis and has been responsible for several human outbreaks in the United States over the past two decades (29, 30). These strains are also frequently associated with high levels of antibiotic resistance (31–34) and are occasionally capable of causing invasive disease. Although CVD 2005 only induced moderate protection against the *S.* Paratyphi B Java strain CDC00-0301, there was a significant delay in time to death (*P* = 0.008). The rapid time to death in unvaccinated animals and the slightly lower LD_50_ suggest that *S*. Paratyphi B Java is more virulent in mice than *S*. Paratyphi B *sensu stricto*. It is possible that there is a difference in endotoxin between these two biotypes and that the very rapid death in unvaccinated animals challenged with *S*. Paratyphi B Java could be due to toxic shock. Vaccine efficacy against *S.* Paratyphi B Java might be improved with additional vaccine doses.

Interestingly, we have found that the *S*. Paratyphi B CVD 2005 vaccine was not able to protect mice against a lethal infection with *S.* Typhimurium, which shares the same O antigens (1,4,[5],12) as *S*. Paratyphi B. This suggests that immune responses against the *S*. Paratyphi B O antigen do not provide cross-protection. Therefore, the cross-protection that we observed against *S*. Paratyphi B Java is most likely due to immune responses generated against shared peptide antigens. Our comparative genomics analyses showed that there is high genetic similarity between *S*. Paratyphi B *sensu stricto* and Java.

In conclusion, *S.* Paratyphi B strain CVD 2005 is a promising candidate for inclusion in a combined typhoid-paratyphoid live attenuated vaccine. This vaccine strain may also provide protection against *S.* Paratyphi B Java disease. Finally, by investigating the commonalities and differences between Salmonella serovars, we can better understand the mechanisms that underpin their different disease pathologies.

## MATERIALS AND METHODS

### Bacterial strains and plasmids.

Isolates used for whole-genome sequencing are listed in Table S1. Strains and plasmids used in this study are listed in Table S6 in the supplemental material. *S.* Paratyphi B, *S*. Paratyphi A, and *S*. Typhi strains were originally isolated from adults who presented with enteric fever symptoms in Santiago, Chile, in the 1980s. *S*. Paratyphi B strains were typed as Java or *sensu stricto* based on their ability to ferment d-tartrate. (Java strains are able to ferment d-tartrate, whereas *sensu stricto* strains do not.) We also confirmed the presence or absence of an intact d-tartrate fermentation gene by PCR as previously described (35). Bacteria were grown in animal-product-free Hy-Soy (HS) medium (1% [wt/vol] soytone [Teknova, Hollister, CA], 0.5% [wt/vol] Hy-Yest [Kerry Bioscience, Beloit, WI], 0.5% [wt/vol] NaCl [American Bio, Natick, MA]) at 37°C. Bacterial strains carrying Δ*guaBA* mutations were grown on media supplemented with 0.005% (wt/vol) guanine.

10.1128/mSphere.00474-18.7 TABLE S6 Bacterial strains and plasmids. Download Table S6, DOCX file, 0.02 MB. Copyright © 2018 Higginson et al. 2018 Higginson et al. This content is distributed under the terms of the Creative Commons Attribution 4.0 International license.

### Whole-genome sequencing.

Bacterial DNA was isolated from *S.* Typhi, *S*. Paratyphi A, and *S*. Paratyphi B isolates (Table S1) using the GenElute bacterial genomic DNA kit (Sigma-Aldrich). Paired-end 100-bp sequencing was performed on the Illumina HiSeq platform by the Institute for Genome Sciences at the University of Maryland, Baltimore.

### Comparative genomics analyses.

Differences in the gene content of the Salmonella genomes were analyzed using *de novo* large-scale BLAST score ratio (LS-BSR) analysis, as previously described (36). The protein-coding genes were predicted in each genome using Prodigal (37) and were grouped into gene clusters with ≥90% nucleotide identity using cd-hit v.4.6 (38). The gene clusters were identified in each of the genomes by comparing their translated peptide sequences against each genome using TBLASTN and generating a BSR value by dividing the bit score of the top hit by the bit score of the same gene cluster sequence compared to itself. Cutoffs of BSR of ≥0.8 and <0.4 were used to identify highly conserved or absent gene clusters, respectively.

The whole-genome phylogeny was generated using a single nucleotide polymorphism (SNP)-based approach as previously described (39, 40). The *In Silico* Genotyper (ISG) (41) was used to identify SNPs in the genomes analyzed in this study, compared to the genome of *S.* Typhimurium strain SL1344 (GenBank accession no. NC_016810.1) as a reference. A total of 86,999 conserved SNP sites, which were identified in all of the genomes analyzed, were used to infer a maximum likelihood using RAxML v.8.2.10 with the generalized time-reversible (GTR) model of nucleotide substitution, the GAMMA model of rate heterogeneity, and 100 bootstrap replicates (42). The final phylogeny image was visualized and labeled using the interactive tree of life software (iTOL) v.3 (43).

### Genetic engineering of *Salmonella* Paratyphi B.

Deletion mutations were created in *S.* Paratyphi B *sensu stricto* strain CMF 6999 by using the lambda red recombinase method (44). Insertion constructs were created by overlapping PCR as previously described (19), using 500-bp flanking regions homologous to the gene of interest, linked to the kanamycin cassette from plasmid pKD13. Linear DNA amplicons were transformed into *S.* Paratyphi B strain CMF 6999, harboring the recombinase plasmid pKD46. After insertion of the kanamycin cassette, at least 150 bp of DNA upstream and downstream of the deletion was sequenced to determine if there were additional changes to the genetic sequence. The kanamycin cassette was then removed by using the flippase system (45). Scar regions and the DNA directly flanking the mutation were sequenced to confirm that additional mutations had not been introduced. Single mutants were created by deleting *guaBA* (CVD 2003) and *clpX* (CVD 2004). To create the double mutant vaccine strain, the *clpX* gene was deleted from strain CVD 2003 to result in strain CVD 2005 (CMF 6999 Δ*guaBA* Δ*clpX*). Mutant phenotypes were confirmed by assessing guanine auxotrophy (*guaBA*) and hyperflagellation (*clpX*) and complemented in *trans*. Complementation plasmids expressing these genes had previously been created using homologous *S.* Typhi genes. To make these plasmids, the *clpX* and *guaBA* genes were amplified from *S.* Typhi CVD 908-*htrA* and cloned into expression plasmid pLowBlu (Table S6) (46). We transformed pATGguaBA into CVD 2003, pATGclpX into CVD 2004, and pATGclpXATGguaBA into CVD 2005. Each strain was also transformed with empty pLowBlu plasmid. Primers used for mutagenesis and confirmation of the deletion are shown in Table S7 in the supplemental material.

10.1128/mSphere.00474-18.8 TABLE S7 Primers used in this study. Download Table S7, DOCX file, 0.02 MB. Copyright © 2018 Higginson et al. 2018 Higginson et al. This content is distributed under the terms of the Creative Commons Attribution 4.0 International license.

### Phenotypic assays.

**(i) Bacterial motility assay.** Bacteria were grown overnight in HS medium supplemented with 20 µg/ml chloramphenicol to maintain complementation plasmids and 0.005% guanine to supplement Δ*guaBA* mutants as required. Cultures were stab inoculated onto motility agar (1% [wt/vol] tryptone [Fisher Scientific, Hampton, NH], 0.5% [wt/vol] NaCl [American Bio], 0.4% [wt/vol] bacteriological agar [American Bio]) and incubated at 37°C for 18 h. The diameter of the zone of motility was then measured.

**(ii) Guanine auxotrophy.** Bacterial lawns were created on chemically defined medium (47), with or without the addition of 0.005% (wt/vol) guanine (Sigma-Aldrich, St. Louis, MO). Plates were assessed for growth after incubation at 37°C for 18 h.

### Mouse infection model for *Salmonella* Paratyphi B.

Animal procedures were approved by the Institutional Animal Care and Use Committee at the University of Maryland School of Medicine. All procedures were conducted in full compliance with the Animal Welfare Act and according to the *Guide for the Care and Use of Laboratory Animals* (50) in a fully accredited AALAC facility. *S.* Paratyphi B strains were grown on HS agar and resuspended in sterile phosphate-buffered saline (PBS [Quality Biologicals, Gaithersburg, MD]) to the appropriate concentration. Six- to 8-week-old female BALB/c mice (*n* = 3 per group) were infected intraperitoneally (i.p.) or perorally (p.o.) with 100 µl of bacterial suspension (10^3^ to 10^9^ CFU). Mice were monitored for signs of illness over the course of 28 days and euthanized if they lost greater than 20% of starting body weight or if they fulfilled other alternative endpoint criteria such as extreme lethargy or loss of mobility. Mortality was recorded, and the 50% lethal dose (LD_50_) was calculated using linear regression analysis.

### Mouse immunization and challenge.

Six- to 8-week-old BALB/c mice (*n* = 12 per group) were immunized either i.n. (10^9^ CFU/10 µl) or p.o. (10^9^ CFU/100 µl) on days 0, 28, and 56. Bacteria were prepared as for the infection model. Serum samples were obtained retro-orbitally on days −1, 27, 55, and 82. Mice were challenged i.p. on day 83 with 2 × 10^7^ CFU/100 µl of *S.* Paratyphi B *sensu stricto* strain CMF 6999 or *S.* Paratyphi B Java strain CDC00-0301, or p.o. with 2 × 10^7^ CFU/100 µl of *S.* Typhimurium I77. Animals were monitored for signs of illness for 28 to 30 days as described above, and mortality was recorded.

### Measurement of anti-LPS antibodies.

The levels of anti-lipopolysaccharide (anti-LPS) antibodies in mouse sera were determined by enzyme-linked immunosorbent assays (ELISA). Plates were coated with Salmonella O group B LPS (0.5 µg in PBS) purified from *S.* Typhimurium strain CVD 1925 for 3 h at 37°C and blocked overnight in 10% (wt/vol) skim milk powder (SMP) in PBS. Wells were washed using 0.05% Tween 20 (Sigma-Aldrich) in PBS (PBST). Sera were assayed by serial dilution in 10% SMP in PBST. Bound antibody was detected by using peroxidase-labeled goat anti-mouse IgG (KPL, Inc., Gaithersburg, MD), followed by the addition of 3,3′,5,5′-tetramethylbenzidine (TMB) substrate solution (KPL). ELISA titers were calculated by interpolation on a standard curve as the inverse of the dilution that showed an increase in absorbance of greater than 0.2 over the blank. Seroconversion was defined as a 4-fold increase in antibody titer postimmunization.

### Serum bactericidal antibody assay.

Serum bactericidal antibody (SBA) assays were performed as described by Boyd et al. (48). Sera were diluted in saline and inactivated at 56°C for 20 min. Serial 2-fold dilutions of inactivated sera were created in a 96-well plate. To each well containing 50 µl serum was added 25 µl of 1:2-diluted (12.5%; final concentration) baby rabbit complement (BRC [Pel-Freez Biologicals, Rogers, AR]) and 3 × 10^2^ CFU/10 µl of log-phase-grown bacterial culture. Plates were incubated at 37°C at 115 rpm for 1 h. The SBA titer was defined as the reciprocal of the highest titer that produced greater than 50% killing, compared to negative-control wells containing bacteria and complement without sera.

### Opsonophagocytosis antibody assay.

The opsonophagocytic capability of sera was assayed by using the method of Ramachandran et al. (49). Briefly, HL-60 cells were propagated in RPMI 1640 (Corning CellGro, Manassas, VA) supplemented with 10% fetal bovine serum (FBS; HyClone, Waltham, MA) and 1% GlutaMax, 1% sodium pyruvate, and 1% penicillin-streptomycin (Life Technologies, Carlsbad, CA). Cells were differentiated with 0.8% dimethyl formamide (DMF [Fisher Scientific]) for 6 days to produce phagocytic neutrophils. Mouse sera and bacteria were prepared as for the SBA. To each well was added 25 µl serum and 3 × 10^2^ CFU of bacteria suspended in 10 µl PBS. Opsonization was allowed to occur for 15 min at 37°C in 5% CO_2_, after which 25 µl BRC and 40 µl differentiated HL-60 cells (4 × 10^4^ cells/well) were added. Plates were incubated at 37°C for 45 min at 160 rpm. The OPA titer was defined as the reciprocal of the highest titer that produced greater than 50% killing compared to negative-control wells (no sera).

### Statistical analyses.

Statistical analysis for phenotypic assays was performed using Student's *t* test (two tailed). To determine statistical significance of antibody responses, Student's *t* test was applied to log_2_-transformed titers. Statistical significance of seroconversion rates and vaccine efficacy were calculated by using Fisher’s exact test (two tailed). All statistical tests were considered significant at a *P* value of <0.05.

### Accession number(s).

Sequences were deposited in the NCBI GenBank database under BioProject ID PRJNA451499.

## References

[1] Buckle GC, Walker CL, Black RE 2012 Typhoid fever and paratyphoid fever: systematic review to estimate global morbidity and mortality for 2010. J Glob Health 2:010401. doi:10.7189/jogh.02.010401. 23198130 PMC3484760

[2] Crump JA, Luby SP, Mintz ED 2004 The global burden of typhoid fever. Bull World Health Organ 82:346–353. 15298225 PMC2622843

[3] Ochiai RL, Wang X, von Seidlein L, Yang J, Bhutta ZA, Bhattacharya SK, Agtini M, Deen JL, Wain J, Kim DR, Ali M, Acosta CJ, Jodar L, Clemens JD 2005 *Salmonella* Paratyphi A rates, Asia. Emerg Infect Dis 11:1764–1766. doi:10.3201/eid1111.050168. 16318734 PMC3367370

[4] Sood S, Kapil A, Dash N, Das BK, Goel V, Seth P 1999 Paratyphoid fever in India: an emerging problem. Emerg Infect Dis 5:483–484. doi:10.3201/eid0503.990329. 10341194 PMC2640769

[5] Woods CW, Murdoch DR, Zimmerman MD, Glover WA, Basnyat B, Wolf L, Belbase RH, Reller LB 2006 Emergence of *Salmonella enterica* serotype Paratyphi A as a major cause of enteric fever in Kathmandu, Nepal. Trans R Soc Trop Med Hyg 100:1063–1067. doi:10.1016/j.trstmh.2005.12.011. 16714040

[6] MacLennan CA, Martin LB, Micoli F 2014 Vaccines against invasive *Salmonella* disease: current status and future directions. Hum Vaccin Immunother 10:1478–1493. doi:10.4161/hv.29054. 24804797 PMC4185946

[7] Black RE, Levine MM, Ferreccio C, Clements ML, Lanata C, Rooney J, Germanier R 1990 Efficacy of one or two doses of Ty21a *Salmonella* Typhi vaccine in enteric-coated capsules in a controlled field trial. Chilean Typhoid Committee. Vaccine 8:81–84. doi:10.1016/0264-410X(90)90183-M. 2180234

[8] Levine MM, Ferreccio C, Black RE, Germanier R 1987 Large-scale field trial of Ty21a live oral typhoid vaccine in enteric-coated capsule formulation. Lancet i:1049–1052. doi:10.1016/S0140-6736(87)90480-6. 2883393

[9] Levine MM, Ferreccio C, Black RE, Lagos R, San Martin O, Blackwelder WC 2007 Ty21a live oral typhoid vaccine and prevention of paratyphoid fever caused by *Salmonella enterica* serovar Paratyphi B. Clin Infect Dis 45 (Suppl 1):S24–S28. doi:10.1086/518141. 17582564

[10] Simanjuntak CH, Paleologo FP, Punjabi NH, Darmowigoto R, Soeprawoto, Totosudirjo H, Haryanto P, Suprijanto E, Witham ND, Hoffman SL 1991 Oral immunisation against typhoid fever in Indonesia with Ty21a vaccine. Lancet 338:1055–1059. doi:10.1016/0140-6736(91)91910-M. 1681365

[11] Pakkanen SH, Kantele JM, Kantele A 2012 Cross-reactive gut-directed immune response against *Salmonella enterica* serovar Paratyphi A and B in typhoid fever and after oral Ty21a typhoid vaccination. Vaccine 30:6047–6053. doi:10.1016/j.vaccine.2012.07.051. 22858557

[12] Wahid R, Simon R, Zafar SJ, Levine MM, Sztein MB 2012 Live oral typhoid vaccine Ty21a induces cross-reactive humoral immune responses against *Salmonella enterica* serovar Paratyphi A and *S.* Paratyphi B in humans. Clin Vaccine Immunol 19:825–834. doi:10.1128/CVI.00058-12. 22492745 PMC3370435

[13] Wahid R, Fresnay S, Levine MM, Sztein MB 2015 Immunization with Ty21a live oral typhoid vaccine elicits crossreactive multifunctional CD8^+^ T-cell responses against *Salmonella enterica* serovar Typhi, *S*. Paratyphi A, and *S*. Paratyphi B in humans. Mucosal Immunol 8:1349–1359. doi:10.1038/mi.2015.24. 25872480 PMC4607552

[14] Reference deleted.

[15] Martin LB, Simon R, MacLennan CA, Tennant SM, Sahastrabuddhe S, Khan MI 2016 Status of paratyphoid fever vaccine research and development. Vaccine 34:2900–2902. doi:10.1016/j.vaccine.2016.03.106. 27083427

[16] Barker RM, Kearney GM, Nicholson P, Blair AL, Porter RC, Crichton PB 1988 Types of *Salmonella* Paratyphi B and their phylogenetic significance. J Med Microbiol 26:285–293. doi:10.1099/00222615-26-4-285. 2456390

[17] Prager R, Rabsch W, Streckel W, Voigt W, Tietze E, Tschape H 2003 Molecular properties of *Salmonella enterica* serotype paratyphi B distinguish between its systemic and its enteric pathovars. J Clin Microbiol 41:4270–4278. doi:10.1128/JCM.41.9.4270-4278.2003. 12958256 PMC193782

[18] Connor TR, Owen SV, Langridge G, Connell S, Nair S, Reuter S, Dallman TJ, Corander J, Tabing KC, Le Hello S, Fookes M, Doublet B, Zhou Z, Feltwell T, Ellington MJ, Herrera S, Gilmour M, Cloeckaert A, Achtman M, Parkhill J, Wain J, De Pinna E, Weill FX, Peters T, Thomson N 2016 What's in a name? Species-wide whole-genome sequencing resolves invasive and noninvasive lineages of *Salmonella enterica* serotype Paratyphi B. mBio 7:e00527-16. doi:10.1128/mBio.00527-16. 27555304 PMC4999539

[19] Tennant SM, Wang JY, Galen JE, Simon R, Pasetti MF, Gat O, Levine MM 2011 Engineering and preclinical evaluation of attenuated nontyphoidal *Salmonella* strains serving as live oral vaccines and as reagent strains. Infect Immun 79:4175–4185. doi:10.1128/IAI.05278-11. 21807911 PMC3187273

[20] Tomoyasu T, Ohkishi T, Ukyo Y, Tokumitsu A, Takaya A, Suzuki M, Sekiya K, Matsui H, Kutsukake K, Yamamoto T 2002 The ClpXP ATP-dependent protease regulates flagellum synthesis in *Salmonella enterica* serovar Typhimurium. J Bacteriol 184:645–653. doi:10.1128/JB.184.3.645-653.2002. 11790733 PMC139528

[21] Tomoyasu T, Takaya A, Isogai E, Yamamoto T 2003 Turnover of FlhD and FlhC, master regulator proteins for *Salmonella* flagellum biogenesis, by the ATP-dependent ClpXP protease. Mol Microbiol 48:443–452. doi:10.1046/j.1365-2958.2003.03437.x. 12675803

[22] Buttle G, Parish H, McLeod M, Stephenson D 1937 The chemotherapy of typhoid and some other non-streptococcal infections in mice. Lancet 229:681–685. doi:10.1016/S0140-6736(00)83397-8.

[23] Hone DM, Harris AM, Chatfield S, Dougan G, Levine MM 1991 Construction of genetically defined double aro mutants of *Salmonella typhi*. Vaccine 9:810–816. doi:10.1016/0264-410X(91)90218-U. 1759503

[24] Powell CJ Jr, DeSett CR, Lowenthal JP, Berman S 1980 The effect of adding iron to mucin on the enhancement of virulence for mice of *Salmonella typhi* strain TY 2. J Biol Stand 8:79–85. doi:10.1016/S0092-1157(80)80049-7. 6156943

[25] Wang JY, Pasetti MF, Noriega FR, Anderson RJ, Wasserman SS, Galen JE, Sztein MB, Levine MM 2001 Construction, genotypic and phenotypic characterization, and immunogenicity of attenuated Δ*guaBA Salmonella enterica* serovar Typhi strain CVD 915. Infect Immun 69:4734–4741. doi:10.1128/IAI.69.8.4734-4741.2001. 11447145 PMC98559

[26] Holt KE, Parkhill J, Mazzoni CJ, Roumagnac P, Weill FX, Goodhead I, Rance R, Baker S, Maskell DJ, Wain J, Dolecek C, Achtman M, Dougan G 2008 High-throughput sequencing provides insights into genome variation and evolution in *Salmonella* Typhi. Nat Genet 40:987–993. doi:10.1038/ng.195. 18660809 PMC2652037

[27] Gat O, Galen JE, Tennant S, Simon R, Blackwelder WC, Silverman DJ, Pasetti MF, Levine MM 2011 Cell-associated flagella enhance the protection conferred by mucosally-administered attenuated *Salmonella* Paratyphi A vaccines. PLoS Negl Trop Dis 5:e1373. doi:10.1371/journal.pntd.0001373. 22069504 PMC3206010

[28] Pasetti MF, Pickett TE, Levine MM, Sztein MB 2000 A comparison of immunogenicity and in vivo distribution of *Salmonella enterica* serovar Typhi and Typhimurium live vector vaccines delivered by mucosal routes in the murine model. Vaccine 18:3208–3213. doi:10.1016/S0264-410X(00)00142-0. 10869765

[29] Denny J, Threlfall J, Takkinen J, Lofdahl S, Westrell T, Varela C, Adak B, Boxall N, Ethelberg S, Torpdahl M, Straetemans M, van Pelt W 2007 Multinational *Salmonella* Paratyphi B variant Java (*Salmonella* Java) outbreak, August–December 2007. Euro Surveill 12:E071220.2 https://www.eurosurveillance.org/content/10.2807/esw.12.51.03332-en. 10.2807/esw.12.51.03332-en 18179762

[30] Griese SE, Fleischauer AT, MacFarquhar JK, Moore Z, Harrelson C, Valiani A, Morrison SE, Sweat D, Maillard JM, Griffin D, Springer D, Mikoleit M, Newton AE, Jackson B, Nguyen TA, Bosch S, Davies M 2013 Gastroenteritis outbreak associated with unpasteurized tempeh, North Carolina, USA. Emerg Infect Dis 19:1514–1517. doi:10.3201/eid1909.130334. 23965530 PMC3810924

[31] Levings RS, Lightfoot D, Hall RM, Djordjevic SP 2006 Aquariums as reservoirs for multidrug-resistant *Salmonella* Paratyphi B. Emerg Infect Dis 12:507–510. doi:10.3201/eid1205.051085. 16704796 PMC3291456

[32] Mulvey MR, Boyd D, Cloeckaert A, Ahmed R, Ng LK 2004 Emergence of multidrug-resistant *Salmonella* Paratyphi B dT+, Canada. Emerg Infect Dis 10:1307–1310. doi:10.3201/eid1007.030862. 15324556 PMC3323318

[33] Threlfall J, Levent B, Hopkins KL, de Pinna E, Ward LR, Brown DJ 2005 Multidrug-resistant *Salmonella* Java. Emerg Infect Dis 11:170–171. doi:10.3201/eid1101.031092. 15714662 PMC3294332

[34] Weill FX, Fabre L, Grandry B, Grimont PA, Casin I 2005 Multiple-antibiotic resistance in *Salmonella enterica* serotype Paratyphi B isolates collected in France between 2000 and 2003 is due mainly to strains harboring *Salmonella* genomic islands 1, 1-B, and 1-C. Antimicrob Agents Chemother 49:2793–2801. doi:10.1128/AAC.49.7.2793-2801.2005. 15980351 PMC1168691

[35] Levy H, Diallo S, Tennant SM, Livio S, Sow SO, Tapia M, Fields PI, Mikoleit M, Tamboura B, Kotloff KL, Lagos R, Nataro JP, Galen JE, Levine MM 2008 PCR method to identify *Salmonella enterica* serovars Typhi, Paratyphi A, and Paratyphi B among *Salmonella* isolates from the blood of patients with clinical enteric fever. J Clin Microbiol 46:1861–1866. doi:10.1128/JCM.00109-08. 18367574 PMC2395068

[36] Sahl JW, Caporaso JG, Rasko DA, Keim P 2014 The large-scale BLAST score ratio (LS-BSR) pipeline: a method to rapidly compare genetic content between bacterial genomes. PeerJ 2:e332. doi:10.7717/peerj.332. 24749011 PMC3976120

[37] Hyatt D, Chen GL, Locascio PF, Land ML, Larimer FW, Hauser LJ 2010 Prodigal: prokaryotic gene recognition and translation initiation site identification. BMC Bioinformatics 11:119. doi:10.1186/1471-2105-11-119. 20211023 PMC2848648

[38] Fu L, Niu B, Zhu Z, Wu S, Li W 2012 CD-HIT: accelerated for clustering the next-generation sequencing data. Bioinformatics 28:3150–3152. doi:10.1093/bioinformatics/bts565. 23060610 PMC3516142

[39] Hazen TH, Sahl JW, Fraser CM, Donnenberg MS, Scheutz F, Rasko DA 2013 Refining the pathovar paradigm via phylogenomics of the attaching and effacing *Escherichia coli*. Proc Natl Acad Sci U S A 110:12810–12815. doi:10.1073/pnas.1306836110. 23858472 PMC3732946

[40] Sahl JW, Steinsland H, Redman JC, Angiuoli SV, Nataro JP, Sommerfelt H, Rasko DA 2011 A comparative genomic analysis of diverse clonal types of enterotoxigenic *Escherichia coli* reveals pathovar-specific conservation. Infect Immun 79:950–960. doi:10.1128/IAI.00932-10. 21078854 PMC3028850

[41] Sahl JW, Beckstrom-Sternberg SM, Babic-Sternberg J, Gillece JD, Hepp CM, Auerbach RK, Tembe W, Wagner DM, Keim PS, Pearson T 2015 The In Silico Genotyper (ISG): an open-source pipeline to rapidly identify and annotate nucleotide variants for comparative genomics applications. bioRxiv 015578. doi:10.1101/015578.

[42] Stamatakis A 2014 RAxML version 8: a tool for phylogenetic analysis and post-analysis of large phylogenies. Bioinformatics 30:1312–1313. doi:10.1093/bioinformatics/btu033. 24451623 PMC3998144

[43] Letunic I, Bork P 2016 Interactive tree of life (iTOL) v3: an online tool for the display and annotation of phylogenetic and other trees. Nucleic Acids Res 44:W242–W245. doi:10.1093/nar/gkw290. 27095192 PMC4987883

[44] Datsenko KA, Wanner BL 2000 One-step inactivation of chromosomal genes in *Escherichia coli* K-12 using PCR products. Proc Natl Acad Sci U S A 97:6640–6645. doi:10.1073/pnas.120163297. 10829079 PMC18686

[45] Cherepanov PP, Wackernagel W 1995 Gene disruption in *Escherichia coli*: TcR and KmR cassettes with the option of Flp-catalyzed excision of the antibiotic-resistance determinant. Gene 158:9–14. doi:10.1016/0378-1119(95)00193-A. 7789817

[46] Vindurampulle C, Barry EM, Levine MM, Galen J July 2013 Attenuated Salmonella enterica serovar Paratyphi A and uses thereof. US patent 8,475,810.

[47] Micoli F, Rondini S, Gavini M, Pisoni I, Lanzilao L, Colucci AM, Giannelli C, Pippi F, Sollai L, Pinto V, Berti F, MacLennan CA, Martin LB, Saul A 2013 A scalable method for O-antigen purification applied to various *Salmonella* serovars. Anal Biochem 434:136–145. doi:10.1016/j.ab.2012.10.038. 23142430 PMC3967520

[48] Boyd MA, Tennant SM, Saague VA, Simon R, Muhsen K, Ramachandran G, Cross AS, Galen JE, Pasetti MF, Levine MM 2014 Serum bactericidal assays to evaluate typhoidal and nontyphoidal *Salmonella* vaccines. Clin Vaccine Immunol 21:712–721. doi:10.1128/CVI.00115-14. 24623629 PMC4018884

[49] Ramachandran G, Boyd MA, MacSwords J, Higginson EE, Simon R, Galen JE, Pasetti MF, Levine MM, Tennant SM 2016 Opsonophagocytic assay to evaluate immunogenicity of nontyphoidal *Salmonella* vaccines. Clin Vaccine Immunol 23:520–523. doi:10.1128/CVI.00106-16. 27030587 PMC4895007

[50] National Research Council. 2011 Guide for the care and use of laboratory animals, 8th ed. National Academies Press, Washington, DC.

