# Supplementary material for: Improving Our Understanding of Salmonella enterica Serovar Paratyphi B through the Engineering and Testing of a Live Attenuated Vaccine Strain
Source: mSphere. 2018 Nov 28;3(6):e00474-18. doi: 10.1128/mSphere.00474-18 (PMC6262260; doi:10.1128/mSphere.00474-18)
Supplement: TABLE S1 [file sph006182708st1.docx]

**TABLE S1 *Salmonella* strains used for whole genome sequencing and analysis**

| Serovar | Phylogroup | Strain ID | Accession no. |
| --- | --- | --- | --- |
| Paratyphi B *sensu stricto* | PG1 | BAS 9 | QGFK00000000 |
|  |  | CMF 6999 | QGFH00000000 |
|  |  | DB 7403 | QGFG00000000 |
|  |  | ELB 6380 | QGFD00000000 |
|  |  | FTP 81 | QGFA00000000 |
|  |  | JSV 7371 | QGEW00000000 |
|  |  | MNZ 6203 | QGEU00000000 |
|  |  | MUG 6222 | QGES00000000 |
|  |  | PMP 2097 | QGEP00000000 |
|  |  | RML 7856 | QGEN00000000 |
|  |  | RPO 6464 | QGEM00000000 |
|  |  | 5644 | QGFS00000000 |
|  |  | 6198 | QGFR00000000 |
|  |  | 7020 | QGFQ00000000 |
|  |  | 11893 2 65 | ERR460158 |
|  |  | 11893 2 66 | ERR460159 |
|  |  | 4502 3 1 | ERR023396 |
| Paratyphi B Java | PG2 | 11893 2 35 | ERR460129 |
|  | PG3 | 11893 2 51 | ERR460144 |
|  | PG4 | 9471 8 16 | ERR278712 |
|  | PG5 | 7688 1 87 | ERR126107 |
|  | PG4 | SPB7 | CP000886.1 |
| Paratyphi A |  | ACE 59 | QGFO00000000 |
|  |  | AFA 115 | QGFN00000000 |
|  |  | GOA 1576 | QGEY00000000 |
|  |  | ATP 4104 | QGFM00000000 |
|  |  | BAL 4481 | QGFL00000000 |
|  |  | BHR 6 | QGFJ00000000 |
|  |  | EAR 6473 | QGFF00000000 |
|  |  | GBC 1563 | QGEZ00000000 |
|  |  | HQA 835 | QGEX00000000 |
|  |  | MRC 7813 | QGET00000000 |
|  |  | VOM 6681 | QGEJ00000000 |
|  |  | AKU_12601 | FM200053 |
|  |  | ATCC 9150 | NC_006511.1 |
| Typhi |  | ABL 114 | QGFP00000000 |
|  |  | CBS 152 | QGFI00000000 |
|  |  | EBC 6494 | QGFE00000000 |
|  |  | ENC 3088 | QGFC00000000 |
|  |  | FCG 7360 | QGFB00000000 |
|  |  | MBM 1277 | QGEV00000000 |
|  |  | OMJ 145 | QGEQ00000000 |
|  |  | POV 7840 | QGEO00000000 |
|  |  | SGC 6022 | QGEL00000000 |
|  |  | TMF 285 | QGEK00000000 |
|  |  | NSS 1317 | QGER00000000 |
|  |  | VTP 6619 | QGEI00000000 |
|  |  | Ty2 | AE014613.1 |
|  |  | CT18 | NC_003198.1 |
|  |  | H58 | NZ_CDLI00000000.1 |
| Typhimurium |  | LT2 | NC_003197.1 |
|  |  | SL1344 | NC_016810.1 |
|  |  | D23580 | NC_016854.1 |
| Enteritidis |  | P125109 | NC_011294.1 |
