# Supplementary material for: Improving Our Understanding of Salmonella enterica Serovar Paratyphi B through the Engineering and Testing of a Live Attenuated Vaccine Strain
Source: mSphere. 2018 Nov 28;3(6):e00474-18. doi: 10.1128/mSphere.00474-18 (PMC6262260; doi:10.1128/mSphere.00474-18)
Supplement: TABLE S4 [file sph006182708st4.docx]

**TABLE S4 Guanine auxotrophy of *guaBA* mutant strains**

|  | **Growth on chemically-defined medium** | |
| --- | --- | --- |
| **Strain** | **Without guanine** | **With guanine** |
| CMF 6999 | + | + |
| CMF 6999 (pLowBlu) | + | + |
| CVD 2003 | - | + |
| CVD 2003 (pLowBlu) | - | + |
| CVD 2003 (pATGguaBA) | + | + |
| CVD 2005 | - | + |
| CVD 2005 (pLowBlu) | - | + |
| CVD 2005 (pATGguaBAATGclpX) | + | + |
