# Supplementary material for: Improving Our Understanding of Salmonella enterica Serovar Paratyphi B through the Engineering and Testing of a Live Attenuated Vaccine Strain
Source: mSphere. 2018 Nov 28;3(6):e00474-18. doi: 10.1128/mSphere.00474-18 (PMC6262260; doi:10.1128/mSphere.00474-18)
Supplement: FIG S1 [file sph006182708sf1.pdf]

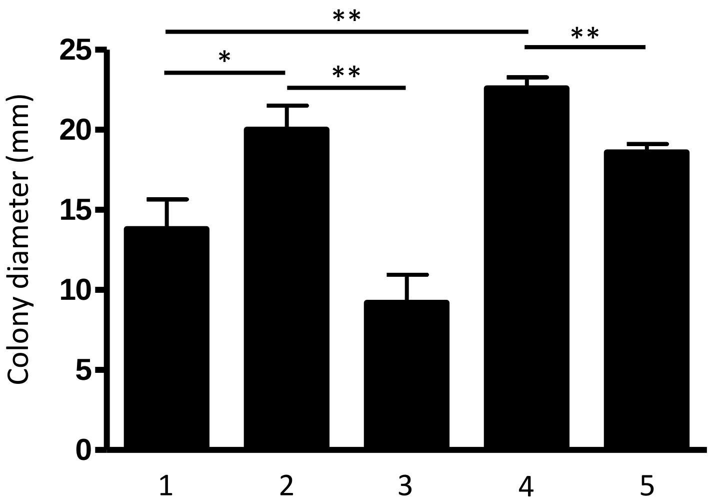

Figure S1. Motility of *S*. Paratyphi B Δ*clpX* mutant strains. Bacterial strains from overnight cultures were inoculated onto motility agar plates by the use of a straight wire. Plates were incubated for 18 h at 37°C, and the zones of motility measured. 1, CMF 6999 (pLowBlu); 2, CVD 2004 (pLowBlu); 3, CVD 2004 (pATGclpX); 4, CVD 2005 (pLowBlu); 5, CVD 2005 (pATGguaBAATGclpX). Data is shown as mean ± SD. Student's t-test. * $p < 0.05$, ** $p < 0.01$
