# Supplementary material for: Improving Our Understanding of Salmonella enterica Serovar Paratyphi B through the Engineering and Testing of a Live Attenuated Vaccine Strain
Source: mSphere. 2018 Nov 28;3(6):e00474-18. doi: 10.1128/mSphere.00474-18 (PMC6262260; doi:10.1128/mSphere.00474-18)
Supplement: TABLE S6 [file sph006182708st6.docx]

**TABLE S6 Bacterial strains and plasmids**

| **Strain or plasmid** | **Details** | **Reference** |
| --- | --- | --- |
| CMF 6999 | *Salmonella* Paratyphi B *sensu stricto*; 1983, Chile | ([1-3](#_ENREF_1)) |
| ELB 6380 | *Salmonella* Paratyphi B *sensu stricto*; 1983, Chile | ([1-3](#_ENREF_1)) |
| JSV 7371 | *Salmonella* Paratyphi B *sensu stricto*; 1983, Chile | ([1-3](#_ENREF_1)) |
| CDC 00-0301 | *Salmonella* Paratyphi B Java, reference strain | ([4](#_ENREF_4)) |
| CDC 01-0516 | *Salmonella* Paratyphi B Java; reference strain | ([4](#_ENREF_4)) |
| CDC 03-0451 | *Salmonella* Paratyphi B Java; reference strain | ([4](#_ENREF_4)) |
| CVD 2003 | *Salmonella* Paratyphi B CMF 6999 ∆*guaBA* | This study |
| CVD 2004 | *Salmonella* Paratyphi B CMF 6999 ∆*clpX* | This study |
| CVD 2005 | *Salmonella* Paratyphi B CMF 6999 ∆*guaBA* ∆*clpX* | This study |
| I77 | *Salmonella* Typhimurium I77; Mali | ([5](#_ENREF_5)) |
| CVD 1925 | *Salmonella* Typhimurium I77 ∆*guaBA* ∆*clpX* | ([6](#_ENREF_6)) |
| pKD13 | FRT-flanked Km^R^ gene, Km^R^, Ap^R^ | ([7](#_ENREF_7)) |
| pKD46 | λ-red recombination vector, *ori* R101, *repA*101ts, P_BAD_-λ red, Ap^R^ | ([7](#_ENREF_7)) |
| pCP20 | Flippase vector, *ori* R101, *repA*101ts, *flp*, Ap^R^, Cm^R^ | ([8](#_ENREF_8)) |
| pLowBlu 184 | Expression plasmid, Cm^R^ | ([9](#_ENREF_9)) |
| pATGguaBA | pLowBlu 184 expressing the *guaBA* gene from *S*. Typhi CVD 908-*htrA* | ([9](#_ENREF_9)) |
| pATGclpX | pLowBlu 184 expressing the *clpX* gene from *S*. Typhi CVD 908-*htrA* | ([9](#_ENREF_9)) |
| pATGclpXATGguaBA | pLowBlu 184 expressing the *guaBA* and *clpX* genes from *S*. Typhi CVD 908-*htrA* | ([9](#_ENREF_9)) |

**REFERENCES**

1. Black RE, Levine MM, Ferreccio C, Clements ML, Lanata C, Rooney J, Germanier R. 1990. Efficacy of one or two doses of Ty21a *Salmonella* Typhi vaccine in enteric-coated capsules in a controlled field trial. Chilean Typhoid Committee. Vaccine 8:81-4.

2. Levine MM, Ferreccio C, Black RE, Germanier R. 1987. Large-scale field trial of Ty21a live oral typhoid vaccine in enteric-coated capsule formulation. Lancet 1:1049-52.

3. Levine MM, Ferreccio C, Black RE, Lagos R, San Martin O, Blackwelder WC. 2007. Ty21a live oral typhoid vaccine and prevention of paratyphoid fever caused by *Salmonella enterica* serovar Paratyphi B. Clin Infect Dis 45 Suppl 1:S24-8.

4. Levy H, Diallo S, Tennant SM, Livio S, Sow SO, Tapia M, Fields PI, Mikoleit M, Tamboura B, Kotloff KL, Lagos R, Nataro JP, Galen JE, Levine MM. 2008. PCR method to identify *Salmonella enterica* serovars Typhi, Paratyphi A, and Paratyphi B among *Salmonella* Isolates from the blood of patients with clinical enteric fever. J Clin Microbiol 46:1861-6.

5. Tapia MD, Tennant SM, Bornstein K, Onwuchekwa U, Tamboura B, Maiga A, Sylla MB, Sissoko S, Kourouma N, Toure A, Malle D, Livio S, Sow SO, Levine MM. 2015. Invasive Nontyphoidal *Salmonella* Infections Among Children in Mali, 2002-2014: Microbiological and Epidemiologic Features Guide Vaccine Development. Clin Infect Dis 61 Suppl 4:S332-8.

6. Tennant SM, Wang JY, Galen JE, Simon R, Pasetti MF, Gat O, Levine MM. 2011. Engineering and preclinical evaluation of attenuated nontyphoidal *Salmonella* strains serving as live oral vaccines and as reagent strains. Infect Immun 79:4175-85.

7. Datsenko KA, Wanner BL. 2000. One-step inactivation of chromosomal genes in *Escherichia coli* K-12 using PCR products. Proc Natl Acad Sci U S A 97:6640-5.

8. Cherepanov PP, Wackernagel W. 1995. Gene disruption in *Escherichia coli*: TcR and KmR cassettes with the option of Flp-catalyzed excision of the antibiotic-resistance determinant. Gene 158:9-14.

9. Vindurampulle C, Barry EM, Levine MM, Galen J. 2013. Attenuated *Salmonella enteric*a serovar Paratyphi A and uses thereof patent 8475810.
