# Supplementary material for: Improving Our Understanding of Salmonella enterica Serovar Paratyphi B through the Engineering and Testing of a Live Attenuated Vaccine Strain
Source: mSphere. 2018 Nov 28;3(6):e00474-18. doi: 10.1128/mSphere.00474-18 (PMC6262260; doi:10.1128/mSphere.00474-18)
Supplement: TABLE S7 [file sph006182708st7.docx]

**TABLE S7 Primers used in this study**

| **Primer name** | **Sequence 5ʹ - 3ʹ** | **Reference** |
| --- | --- | --- |
| guaBAF | CTCGTACAGCGTAATATTGGCG | This study |
| guaBAOR | GAAGCAGCTCCAGCCTACACGGGCAATATCTCACCTGGGAG | This study |
| guaBAOF | GGTCGACGGATCCCCGGAATAGCCGATAATCCTTCCTGTGT | This study |
| guaBAR | TCTTTATCCGCTCACGCTTCA | This study |
| guaBA3 | CCGCGTAAGCCACTAACG | This study |
| guaBA4 | GTGATCACCCCAACGCAG | This study |
| clpXF7 | ATTAAGCCAGACGTCAGCAC | ([1](#_ENREF_1)) |
| clpmutR2 | GAAGCAGCTCCAGCCTACACGAGTCAAAACCTCTTCTTTG | ([1](#_ENREF_1)) |
| clpmutF4 | GGTCGACGGATCCCCGGAATTTAAACATTCATACAATCAGTTAG | ([1](#_ENREF_1)) |
| clpPXR7-SPB7 | AGCACTTCTTGTTCTCGCTC | This study |
| P1 | GTGTAGGCTGGAGCTGCTTC | ([2](#_ENREF_2)) |
| P4 | ATTCCGGGGATCCGTCGACC | ([2](#_ENREF_2)) |

**REFERENCES**

1. Tennant SM, Wang JY, Galen JE, Simon R, Pasetti MF, Gat O, Levine MM. 2011. Engineering and preclinical evaluation of attenuated nontyphoidal *Salmonella* strains serving as live oral vaccines and as reagent strains. Infect Immun 79:4175-85.

2. Datsenko KA, Wanner BL. 2000. One-step inactivation of chromosomal genes in *Escherichia coli* K-12 using PCR products. Proc Natl Acad Sci U S A 97:6640-5.
